# Achievement of weight loss in patients with overweight during dietetic treatment in primary health care

**DOI:** 10.1371/journal.pone.0225065

**Published:** 2019-11-27

**Authors:** Lisa D. M. Verberne, Chantal J. Leemrijse, Markus M. J. Nielen, Roland D. Friele

**Affiliations:** 1 Nivel, Netherlands Institute for Health Services Research, Utrecht, The Netherlands; 2 Tilburg School of Social and Behavioral Sciences, Tilburg University, Tilburg, The Netherlands; University of Alabama at Birmingham, UNITED STATES

## Abstract

**Introduction:**

Dietitians are the preferred primary health care professionals for nutritional care in overweight patients. Guidelines for dietitians recommend a weight reduction of ≥ 5% of initial body weight after one year of treatment. The purpose of this study was to evaluate weight change in patients with overweight who were treated by dietitians in Dutch primary health care, and to identify patient characteristics that were associated with it.

**Materials and methods:**

This observational study data was based on real life practice data of patients with overweight during the period 2013–2017, derived from dietetic practices that participated in the Nivel Primary Care Database. Multilevel linear regression analyses were performed to investigate weight change after dietetic treatment and to explore associations with patient characteristics.

**Results:**

In total, data were evaluated from 56 dietetic practices and 4722 patients with a body mass index (BMI) ≥ 25 kg/m^2^. The mean treatment time was 3 hours within an average timeframe of 5 months. Overall, patients had a mean weight change of -3.5% (95% CI: -3.8; -3.1) of their initial body weight, and a quarter of the patients reached a weight loss of 5% or more, despite the fact that most patients did not meet the recommended treatment duration of at least one year. The mean BMI change was -1.1 kg/m^2^ (95% CI: -1.2; -1.0). Higher weight reductions were shown for patients with a higher initial BMI and for patients with a longer treatment time. Sex and age were not associated with weight change, and patients with other dietetic diagnoses, such as diabetes, hypertension, and hypercholesterolemia, had lower weight reductions.

**Conclusions:**

This study showed that dietetic treatment in primary health care coincided with modest weight reduction in patients with overweight. The weight loss goals were not reached for most patients, which was possibly due to a low treatment adherence.

## Introduction

Worldwide, the prevalence of overweight and obesity has increased over the past three decades and has become a major global health challenge [1]. Estimates for the European region indicate that more than half of the people are overweight or obese (i.e. having a Body Mass Index (BMI) ≥ 25 kg/m^2^) [2]. Overweight, and obesity in particular, is an important risk factor for cardio metabolic diseases, including diabetes mellitus type 2 and cardiovascular diseases, and is also associated with common risk factors for cardiovascular diseases, such as hypertension and hypercholesterolemia [3].

In Europe, almost all primary health care systems provide services for prevention, detection, and management of cardio metabolic diseases [3]. Like in many other European countries, in the Netherlands, dietitians are the preferred primary health care professionals to provide nutritional care to overweight individuals [4, 5]. Dietary and lifestyle modifications are the primary treatment goals for the treatment of overweight patients. Guidelines for dietitians recommend approximately one year of intensive treatment, followed by one year with regular contact for weight maintenance. A weight loss goal of ≥ 10–15% and ≥ 5–10% is advocated after one year of treatment for overweight patients with and without co-morbidities, respectively [6, 7]. Evidence suggests that a weight loss of 3–5% of initial body weight might already lead to clinically meaningful improvements, e.g. on blood glucose levels [8]. However, larger weight losses produce greater benefits, e.g. on blood pressure, and lipid levels [8–10].

A recent review of randomised controlled trials indicated that nutritional care, provided by dietitians in the primary health care system, resulted in improvements in several health outcomes, including weight loss, compared to usual care (not including nutritional care) [11]. Another review also showed that dietetic intervention has significant impacts on several health outcomes in obese patients, and has substantial economic benefits [12].

Most previous studies that have demonstrated the effectiveness of dietetic treatment were interventional trials. Only a few observational studies have been conducted that evaluated real life practice data [13, 14]. The observational study of Tol and colleagues showed that patients’ BMI decreased by 0.94 kg/m^2^ during dietetic treatment in primary health care in the Netherlands [14]. The study was based on routinely recorded data from patients with overweight who had completed dietetic treatment between 2006 and 2012. In recent years, however, several changes have occurred in the reimbursement of primary health care in the Netherlands which might negatively have affected dietetic treatment. An important change was the reduction in the maximum number of hours of dietetic treatment that will be reimbursement by the basic health insurance from four to three hours per calendar year from 2013. Furthermore, the health care costs that have to be paid out-of-pocket by the patient almost doubled between 2012 and 2016, which is associated with patients refraining from accessing medical care [15].

The present study updates previous results on BMI changes during dietetic treatment as reported by Tol et al. [14], with data from patients who started a dietetic treatment between 2013 and 2016. The aim of the study was to assess weight change in patients with overweight who were treated by dietitians, and to identify patient characteristics that were associated with it.

## Materials and methods

### Study design

This observational study was based on routinely recorded data from Dutch dietetic practices that participated in the Nivel Primary Care Database (Nivel-PCD) within the period 2013–2017. The Nivel-PCD contains anonymised patient data from electronic health records, which is extracted from software programmes of primary care dietetic practices. In the software programme, dietitians record all data needed for reimbursement, e.g. patients age, sex, dietetic diagnoses, treatment time, and dates of consultations (see also Table 1). Furthermore, the software programme allows dietitians to record other relevant information, e.g. anthropometric measurements, level of education, and living situation.

**Table 1:** Explanation of dietetic diagnosis and treatment time.

During the initial visit, the dietitian assesses the medical condition of the patient and records one or more dietetic diagnoses, i.e. (risk factors for) diseases related to the nutritional problem, according to a nationally used coding list. In general, the initial visit takes 45–60 minutes of direct treatment time. Follow-up consultations take 15–30 minutes.

### Study population

All electronic health records were selected from patients who were ≥ 18 years old, who had a recorded dietetic diagnosis of being overweight or obese, and who started a dietetic treatment between January 2013 and December 2016. A treatment was considered to have ended if a reason for ending the treatment was recorded in the software programme used by the dietitian, or if the time elapsed since the last consultation date was more than one year. In order to evaluate weight change during treatment, patients without a recorded weight measurement at start and/or end of the dietetic treatment were excluded. Furthermore, patients were excluded who had a recorded dietetic diagnosis with contraindications for weight loss, such as gestational diabetes.

### Outcome measures

Three outcome measures were defined to evaluate weight change following dietetic treatment, including, “weight change in kilograms (kg)”, “weight change as a percentage of initial body weight”, and “BMI change”. “Weight change in kg” was defined as: body weight at end of treatment–body weight at start of treatment, “Weight change as percentage of initial body weight” was defined as: (body weight at end of treatment–body weight at start of treatment)/body weight at start of treatment*100, and “BMI change” was defined as: BMI at end of treatment–BMI at start of treatment.

### Independent variables

All patient characteristics were selected that were available in Nivel-PCD, might affect weight change, and were recorded for most of the patients (see S1 Table). The selected variables included sex, age, treatment time (categorised), treatment duration (categorised), and a variable that defined whether a patient had other recorded dietetic diagnoses. Therefore, three categories were established: 1) no other recorded dietetic diagnosis; 2) a recorded dietetic diagnosis of diabetes mellitus type 2, hypertension, and/or hypercholesterolemia; and 3) a recorded dietetic diagnosis other than diabetes mellitus type 2, hypertension, or hypercholesterolemia. All independent variables were recorded at the start of the treatment.

### Statistical analyses

Analyses were conducted using Stata 14.2. Descriptive analyses were performed to present patient characteristics. To investigate the mean weight change at the end of the dietetic treatment, three separate multilevel linear regression analyses were performed, including a random intercept to account for clustered data of patients (level 1) within dietetic practices (level 2). Patient characteristics were added to the model with the outcome “weight change as percentage of initial body weight”. The continuous variable age was centred at the mean to improve interpretation of the intercept. Due to the multicollinearity between treatment time and treatment duration, only treatment time was added to the model. All analyses were performed for the total population and for the subgroups of initial BMI-categories (25–30, 30–35, and ≥35 kg/m^2^). Except for six patients, the initial BMI category was specified for all patients. Associations were considered statistically significant if the two-tailed p-value was < 0.05.

### Ethics approval

This study has been approved by the applicable governance bodies of Nivel Primary Care Database (NZR-00317.043). Dutch law allows the use of electronic health records for research purposes under certain conditions. According to this legislation, neither obtaining informed consent from patients nor approval by a medical ethics committee is obligatory for this type of observational study containing no directly identifiable data from the NIVEL PCD (Dutch Civil Law, Article 7:458).

## Results

Initially, 8816 patients were selected in the Nivel-PCD with a recorded dietetic diagnosis of being overweight and having completed treatment. After applying all selection criteria, most patients were excluded due to a missing weight measure at the end of treatment. Finally, 4722 patients who were treated in 56 dietetic practices were included in the current study.

Table 2 shows the characteristics of the study population. The patients had an average age of 55 years, were most likely to be female, and 63% of the patients was obese. The mean treatment time and treatment duration were 3 hours and 5 months, respectively. Patients had on average 4 consultations with their dietitian during treatment. Stratification by initial BMI-category showed that patients with a higher BMI were younger, had other recorded dietetic diagnoses less often, and had a longer treatment time than patients with a lower initial BMI. S2 Table shows the characteristics of the patients that did not meet all the selection criteria. The characteristics of these excluded patients were comparable to those included in the present study, except for treatment time, which was substantially lower in the group of excluded patients.

**Table 2:** Patient characteristics.

	BMI 25–30	BMI 30–35	BMI ≥ 35	Total
Patients, N	1733	1731	1252	4722
Sex, % female	60.1	59.7	69.1	62.3
Mean age in years (SD)	56.2 (15.8)	55.3 (15.0)	51.9 (15.3)	54.7 (15.5)
Mean initial body weight in kg (SD)	82.3 (10.0)	94.4 (11.7)	113.2 (18.9)	95.2 (17.9)
Mean initial BMI (kg/m^2^) (SD)	27.8 (1.4)	32.2 (1.4)	39.4 (4.1)	32.5 (5.2)
Dietetic diagnosis, % patients				
No other diagnosis	31.5	38.6	46.0	38.0
Diagnosis of diabetes mellitus type 2, hypertension, and/or hypercholesterolemia	54.9	52.4	43.4	50.9
A diagnosis other than diabetes mellitus type 2, hypertension, or hypercholesterolemia	13.6	9.0	10.6	11.1
Treatment time, % patients				
≤ 2 hours	29.7	24.4	19.8	25.1
2–3 hours	42.5	39.6	40.6	40.9
> 3 hours	27.8	36.1	39.6	34.0
Treatment duration, % patients				
≤ 6 months	73.7	66.7	68.5	69.7
6–12 months	19.1	23.2	21.4	21.4
> 12 months	7.2	10.1	10.1	9.0

BMI, body mass index.

Table 3 presents measures of weight change at the end of dietetic treatment. Overall, patients with overweight had a mean weight change of -3.5% (95% CI: -3.8; -3.1) of their initial body weight at end of the dietetic treatment, and a quarter of the patients reached a weight loss of ≥ 5%. The mean BMI change was -1.1 kg/m^2^ (95% CI: -1.2; -1.0) and 4% of the total study population reached a BMI < 25 kg/m^2^. The subgroup analyses showed higher weight reductions for patients with a higher initial BMI.

**Table 3:** Weight change at the end of dietetic treatment.

	BMI 25–30	BMI 30–35	BMI ≥ 35	Total
Weight change (kg)	-2.7 (-3.0; -2.4)	-3.3 (-3.8; -2.8)	-4.2 (-4.9; -3.5)	-3.3 (-3.7; -3.0)
Weight change (% of initial body weight)	-3.1 (-3.5; -2.8)	-3.4 (-4.0; -2.9)	-3.7 (-4.3; -3.1)	-3.5 (-3.8; -3.1)
BMI change (kg/m^2^)	-0.9 (-1.0; -0.8)	-1.1 (-1.3; -0.9)	-1.4 (-1.7; -1.2)	-1.1 (-1.2; -1.0)

BMI, body mass index.

Regression coefficients are presented with their 95% confidence interval. A random intercept was included to account for clustered data of patients within dietetic practices.

The results of multilevel regression analyses for the association of patient characteristics with the percentage weight change are presented in Table 4 and show that treatment time was strongly associated with weight loss. Patients with > 3 hours of treatment time had an average weight change of -4.6% of their initial body weight, compared to -2.0% for patients with ≤ 2 hours of treatment time (with mean values for the other independent variables). Furthermore, the results show that patients with one or more other dietetic diagnoses had lower weight reductions than patients with overweight or obesity as their only dietary diagnosis. Sex and age were not associated with weight change.

**Table 4:** Regression coefficients for associations between patient characteristics and weight change (as % of initial body weight).

	BMI 25–30	BMI 30–35	BMI ≥ 35	Total
Sex				
Male (reference)				
Female	0.00 (-0.42; 0.42)	0.23 (-0.24; 0.70)	0.58 (-0.05; 1.21)	0.25 (-0.04; 0.53)
Age (reference mean)	0.00 (-0.01; 0.01)	-0.01 (-0.03; 0.01)	-0.01 (-0.04; 0.01)	-0.01 (-0.02; 0.00)
Dietetic diagnosis				
No other diagnosis (reference)				
Diagnosis of diabetes mellitus type 2, hypertension, and/or hypercholesterolemia,	-0.02 (-0.53; 0.48)	0.51 (-0.03; 1.05)	0.76 (0.09; 1.44) *	0.41 (0.09; 0.74) *
A diagnosis other than diabetes mellitus type 2, hypertension, or hypercholesterolemia.	0.57 (-0.08; 1.21)	0.74 (-0.12; 1.61)	0.79 (-0.20; 1.78)	0.76 (0.29;1.23) *
Treatment time				
≤ 2 hours (reference)				
2–3 hours	-1.44 (-1.90; -0.97)*	-1.53 (-2.12; -0.94)*	-1.26 (-2.05; -0.46)*	-1.39 (-1.73; -1.05)*
> 3 hours	-2.40 (-2.92; -1.88)*	-2.56 (-3.18; -1.95)*	-3.11 (-3.92; -2.30)*	-2.67 (-3.03; -2.31)*
Intercept	-1.92 (-2.54; -1.30)	-2.38 (-3.16; -1.60)	-2.59 (-3.52; -1.67)	-2.41 (-2.89; -1.94

BMI, body mass index.

Regression coefficients are presented with their 95% confidence interval. A random intercept was included to account for clustered data of patients within dietetic practices.

*Significant regression coefficient (P < 0.05)

Intercept: male, mean age, no other dietetic diagnosis, treatment time 0–2 hours.

## Discussion

### Main findings

By evaluating real life practice data from dietitians during the period 2013–2017, this study showed that dietetic treatment in patients with overweight coincided with a mean reduction of 1.1 BMI point, corresponding to a weight loss of 3.5% of initial body weight, with an average treatment time of 3 hours within a timeframe of 5 months.

The results for the BMI change were consistent with the study by Tol et al. [14] that evaluated similar primary health care data over the period 2006–2012. Comparable findings were presented in a study evaluating a lifestyle programme for patients with overweight implemented in Dutch primary health care that reported a mean weight reduction of 0.9 kg/m^2^ one year after the intervention [16]. Higher weight reductions were shown in an observational study from the USA investigating a weight loss programme for patients with obesity in a physician’s office [17]. In this study, 80% of the patients had a weight loss of 5.6% of their initial body weight at end of the follow up. However, the average follow-up was 21 months, which was much longer than the follow-up in our study.

Other studies that have reported weight changes after dietetic treatment were mostly randomised controlled trials. Willaing et al. [18] showed comparable results in a randomised controlled trial implemented in Danish primary health care. Patients at high risk of ischaemic heart disease and with a mean BMI of 33 kg/m^2^ were allocated to a general practitioner or a dietitian to receive nutritional care according to usual practice. After one year, a mean reduction of 1.1 BMI point was found in the dietitian group. Furthermore, a meta-analysis of 46 randomised controlled trials among patients with overweight, comparing nutritional care based weight loss programmes with usual care, showed a mean treatment effect of -1.9 BMI point and a 6% reduction of initial body weight after one year [19]. However, the interventions, study samples, and weight changes of the underlying studies were heterogeneous, and the studies were generally of moderate to poor quality.

The results of our study showed that treatment time was positively associated with weight reduction, which has also been observed in previous studies [13, 14]. Moreover, in accordance with other studies, a higher initial BMI was associated with a higher weight change [13, 14, 20]. This finding can be explained by the fact that people with a higher body weight have a higher basal metabolism, and therefore, can have a higher energy intake than people with a lower weight to lose the same amount of weight [21]. Furthermore, our data show that patients in the highest weight category (BMI ≥ 35) had a longer treatment time, and had less often one or more other dietetic diagnosis, compared to patients in the lower weight categories. Patients with other dietary diagnoses, such as diabetes mellitus type 2 or hypertension, use medication (e.g. sulfonylureas, or beta-blockers) that could negatively influence weight loss [22, 23].

### Strengths and limitations

A major strength of this study is the use of anonymous patient data that are routinely recorded by dietitians in regular dietetic practices. We evaluated data of many patients within a realistic context, without bias from self-reporting. Herewith, our study differs from most other studies that have been conducted in an intervention setting.

A limitation of our study was the data availability, since many dietitians did not routinely record all patient information in their software programme. For many patients, weight was not recorded at the end of treatment, and therefore, these patients could not be included in the present study. Treatment time was substantially lower in the group of excluded patients, and we therefore assume that our study population is not fully representative of the total overweight population that visits dietitians, since it probably includes the more motivated patients. However, data on characteristics of motivation, were not collected in this study.

### Implications of the findings

The present study showed that only a quarter of the patients reached the weight loss goal of ≥ 5% of their initial body weight at end of dietetic treatment in primary health care. Furthermore, this study highlights that most patients did not meet the recommended treatment duration of at least one year, suggesting that many patients quit dietetic treatment prematurely. Our data revealed that only 9% of the patients had a treatment duration of one year or longer. Nevertheless, the treatment duration in the period 2013–2017 was comparable to the treatment duration in the period 2006–2012 [14]. This suggests that the financial changes in primary health care in recent years did not affect the use of dietetic health care.

The value of dietetic treatment is hard to measure if patients do not adhere to the recommended treatment guidelines. Studies evaluating the effectiveness of dietetic treatement in randomised controlled trials indicated a longer follow-up as an important factor to enhance weight loss and reduce weight regain [11]. For further research, it would be interesting to gain more insight into the factors that are associated with quitting dietetic treatment in primary health care.

## Conclusions

This study showed that dietetic treatment in primary health care resulted in modest weight reduction in overweight and obese patients. Furthermore, this study revealed that a longer treatment time and a higher initial BMI were associated with higher weight loss. Weight loss goals were not reached for most patients, which was probably due to a low treatment adherence in the majority of patients.

## Supporting information

S1 Table Completeness of patient information. (DOCX)

S2 Table Patient characteristics of excluded patients. (DOCX)
