# Supplementary material for: Achievement of weight loss in patients with overweight during dietetic treatment in primary health care
Source: PLoS One. 2019 Nov 27;14(11):e0225065. doi: 10.1371/journal.pone.0225065 (PMC6880966; doi:10.1371/journal.pone.0225065)
Supplement: S1 Table — (DOCX) [file pone.0225065.s001.docx]

| Completeness of patient information (N=4722) | | |
| --- | --- | --- |
| Recorded patient characteristics | Completeness (N) | Completeness (%) |
| Sex | 4721 | 99.9 |
| Age | 4722 | 100 |
| Education | 816 | 17.3 |
| Living situation | 2293 | 48.6 |
| Had a previous dietetic treatment | 989 | 20.9 |
| Communication problems | 1004 | 21.3 |
| Psychological problems | 979 | 20.7 |
| Intellectual problems | 996 | 21.1 |
| Dietetic diagnoses (min.1, max.4) | 4722 | 100 |
| Treatment time | 4722 | 100 |
| Treatment duration | 4722 | 100 |
