# Supplementary material for: Achievement of weight loss in patients with overweight during dietetic treatment in primary health care
Source: PLoS One. 2019 Nov 27;14(11):e0225065. doi: 10.1371/journal.pone.0225065 (PMC6880966; doi:10.1371/journal.pone.0225065)
Supplement: S2 Table — (DOCX) [file pone.0225065.s002.docx]

| Characteristics of excluded patients | | | | |
| --- | --- | --- | --- | --- |
|  | BMI 25-30 | BMI 30-35 | BMI ≥35 | Total |
| Patients, N | 1473 | 1328 | 923 | 3809 |
| Sex, % female | 40.3 | 41.2 | 34.3 | 38.9 |
| Mean age in years (SD) | 56.1 (16.4) | 54.7 (15.3) | 51.2 (15.4) | 54.4 (15.9) |
| Mean initial body weight in kg (SD) | 81.3 (10.5) | 94.7 (11.7) | 114.3 (17.5) | 94.2 (18.5) |
| Mean initial BMI (kg/m^2^) (SD) | 27.5 (1.4) | 32.3 (1.4) | 39.4 (4.0) | 32.2 (5.3) |
| Dietetic diagnosis, % patients |  |  |  |  |
| No other diagnosis | 26.1 | 33.7 | 44.6 | 33.9 |
| Diagnosis of diabetes mellitus type 2, hypertension, and/or hypercholesterolemia | 55.0 | 53.1 | 44.5 | 51.4 |
| A diagnosis other than diabetes mellitus type 2, hypertension, or hypercholesterolemia | 18.9 | 13.2 | 10.8 | 14.7 |
| Treatment time, % patients |  |  |  |  |
| ≤ 2 hours | 60.4 | 48.8 | 43.1 | 52.0 |
| 2–3 hours | 22.5 | 25.4 | 27.8 | 24.9 |
| > 3 hours | 17.1 | 25.8 | 29.0 | 23.2 |
| Treatment duration, % patients |  |  |  |  |
| ≤ 6 months | 83.2 | 73.8 | 71.2 | 78.0 |
| 6–12 months | 11.5 | 18.8 | 18.7 | 16.0 |
| > 12 months | 5.2 | 7.4 | 10.1 | 7.3 |
| BMI: body mass index  Excluded patients were those with a missing weight measure at the start and/or the end of treatment and those who had a recorded diagnosis (next to overweight), for which weight loss might not the goal for treatment.  Missing: 85 for initial BMI category, 693 for initial weight, 720 for initial BMI. | | | | |
